# Questions on the new classification of periodontal and preimplantation diseases

**DOI:** 10.34172/japid.2021.012

**Published:** 2021-08-23

**Authors:** Mohammadtaghi Chitsazi, Amirreza Babaloo, Hamidreza Mohammadi

**Affiliations:** ^1^Department of Periodontics, Faculty of Dentistry, Tabriz University of Medical Sciences, Tabriz, Iran

## Background

Armitage’s classification of periodontal diseases came to practice in the 1999 Periodontal Workshop and has been diligently followed ever since.1 In the last two decades, knowledge of periodontal diseases has increased rapidly. A new class of the disease has also been added to the classification of periodontal diseases, with similar pathogenesis, but it arises around dental implants. There was a compelling need to understand and categorize this information about the principles of evidence-based dentistry and reach a worldwide consensus. To achieve this goal, the American Academy of Periodontology and the European Periodontology Federation convened a meeting in November 2017 to organize a workshop entitled “Classification of Periodontal and Peri-implant Diseases” in June 2018 in both journals of Periodontology: a leading journal of the American Academy of Periodontology and the Journal of Clinical Periodontology, a leading journal of the European Periodontology Federation, with 19 review articles and four consensus reports.^2^ Since the diagnosis of patients’ periodontal status is the basis of dental examinations, this classification has affected the entire dental science and community. To begin with, periodontal health was defined as the absence of signs of inflammation indicated by <10% of sites with bleeding on probing and probing depths <3 mm. Gingivitis was defined as a reversible inflammation indicated by bleeding on probing in more than 10% of sites, with probing depths of <3 mm. Gingivitis was further divided depending on whether it is present in an intact periodontium, in a reduced periodontium (e.g., gingival recession cases), or in a successfully treated periodontally stable patient. The primary etiologic factor for the above conditions was considered to be dental biofilm. In contrast, a separate category was assigned to non-dental plaque-induced gingival diseases like viral, bacterial, autoimmune diseases, or numerous other conditions affecting the gingiva not induced by dental plaque.^3^

The most important changes have occurred concerning the classification of periodontitis, in which the staging and grading system of the disease is similar to the system used in oncology to classify cancers. Periodontitis is now no longer divided into two forms: chronic and aggressive; as evidence suggests, it is essentially the same disease process with differing rates of extent, progression, and severity.^4^ The stages of periodontitis, namely I, II, III, and IV, were based on severity and complexity of management.^5^ The extent and distribution were localized, generalized, or molar incisor distribution, and the grades were A, B, or C, denoting the rate of progression, which was either slow, moderate, or rapid, respectively.

Necrotizing periodontal diseases and periodontitis as a manifestation of systemic diseases were classified as other different forms of periodontitis.^6^

One component of this classification has raised some questions about the criteria for “extent” in the staging process.

The question is whether the extent should describe those teeth that harbor the most severe sites that define the patient’s stage or those teeth with any degree of attachment loss.

In view of these criteria, “extent” refers to a stage that indicates the overall severity and complexity of the case. Therefore, post-stage measurement determines the percentage of teeth at the stage intensity level and provides meaningful information for the dentist, as it shows the percentage of teeth that have been most severely affected and possibly has to be treated with more complexity. It is, however, important to define appropriately what is a hopeless tooth (also termed irrational to treat).

Another question is whether to consider lost teeth because of periodontitis and the existing teeth with an evident hopeless prognosis. Hopeless teeth are those in which the attachment loss approximates the apex of the root circumferentially,^2^ in combination with a high degree of tooth hypermobility (degree III).

Another issue with this classification is the lack of treatment options for each condition. In the previous classification, antibiotic treatment strategies were proposed in aggressive narcotizing periodontal diseases, while with the above classification, it would be challenging to provide treatment strategies due to the lack of a clear boundary.

One of the most important criteria of a good therapeutic classification is to determine the clear boundary between the different conditions of a disease and also the possibility of establishing a relationship between the patient’s clinical manifestation and the conditions in the classification. This problem was possible, to some extent, in the previous classification, but in the new classification, due to different criteria, the possibility of adapting the patient’s clinical condition to the above classification is reduced.

The mentioned concerns have been raised as the challenges of the above classification, and finding answers to them will help clarify this classification
